# Resuscitation speed affects brain injury in a large animal model of traumatic brain injury and shock

**DOI:** 10.1186/s13049-014-0046-2

**Published:** 2014-08-14

**Authors:** Martin Sillesen, Guang Jin, Pär I Johansson, Hasan B Alam

**Affiliations:** 1 Department of Surgery, Division of Trauma, Emergency Surgery and Surgical Critical Care, Massachusetts General Hospital/Harvard Medical School, Boston 02114, MA, USA; 2 Department of Surgery, Copenhagen University Hospital, Hillerød. Dyrehavevej 29, Hillerød, 3400, Denmark; 3 Department of Surgery, University of Michigan Hospital, 2920 Taubman Center/5331, 1500 E. Medical Center Drive, Ann Arbor 48109, MI, USA; 4 Capital Region Blood Bank, Copenhagen University Hospital, Rigshospitalet. Blegdamsvej 9, Copenhagen, 2100, Denmark; 5 Department of Surgery, University of Texas Medical School, Houston 77030, TX, USA

**Keywords:** Traumatic brain injury, Hemorrhagic shock resuscitation, Fresh frozen plasma, Swine

## Abstract

**Background:**

Optimal fluid resuscitation strategy following combined traumatic brain injury (TBI) and hemorrhagic shock (HS) remain controversial and the effect of resuscitation infusion speed on outcome is not well known. We have previously reported that bolus infusion of fresh frozen plasma (FFP) protects the brain compared with bolus infusion of 0.9% normal saline (NS). We now hypothesize reducing resuscitation infusion speed through a stepwise infusion speed increment protocol using either FFP or NS would provide neuroprotection compared with a high speed resuscitation protocol.

**Methods:**

23 Yorkshire swine underwent a protocol of computer controlled TBI and 40% hemorrhage. Animals were left in shock (mean arterial pressure of 35 mmHg) for two hours prior to resuscitation with bolus FFP (n = 5, 50 ml/min) or stepwise infusion speed increment FFP (n = 6), bolus NS (n = 5, 165 ml/min) or stepwise infusion speed increment NS (n = 7). Hemodynamic variables over a 6-hour observation phase were recorded. Following euthanasia, brains were harvested and lesion size as well as brain swelling was measured.

**Results:**

Bolus FFP resuscitation resulted in greater brain swelling (22.36 ± 1.03% vs. 15.58 ± 2.52%, p = 0.04), but similar lesion size compared with stepwise resuscitation. This was associated with a lower cardiac output (CO: 4.81 ± 1.50 l/min vs. 5.45 ± 1.14 l/min, p = 0.03). In the NS groups, bolus infusion resulted in both increased brain swelling (37.24 ± 1.63% vs. 26.74 ± 1.33%, p = 0.05) as well as lesion size (3285.44 ± 130.81 mm^3^ vs. 2509.41 ± 297.44 mm^3^, p = 0.04). This was also associated with decreased cardiac output (NS: 4.37 ± 0.12 l/min vs. 6.35 ± 0.10 l/min, p < 0.01).

**Conclusions:**

In this clinically relevant model of combined TBI and HS, stepwise resuscitation protected the brain compared with bolus resuscitation.

## Background

Optimal fluid resuscitation strategies in patients with hemorrhagic shock (HS) remain controversial, although both crystalloids (1) and fresh frozen plasma (FFP) remain key components of both pre and in-hospital resuscitation strategies. Maintaining end-organ oxygenation by ensuring adequate tissue perfusion pressures as well as reducing edema formation by minimizing fluid extravasation is key to the resuscitation success, regardless of fluid choice.

While the choice of resuscitation fluid has been the subject of intense investigation [1],[2], little is known of the effect resuscitation speed. The Advanced Trauma Life Support (ATLS) guidelines place emphasis on early restoration of adequate tissue perfusion by rapid infusion of crystalloids [3], thus theoretically minimizing the time to adequate end organ oxygenation. In contrast, several studies have indicated that rapid bolus infusion of crystalloids and artificial colloids may be associated with lower post resuscitation systemic blood pressures and higher mortality compared with slow or stepwise infusion in general trauma [4]–[6]. If higher infusion speeds are indeed associated with higher levels of fluid extravasation, this may be particularly detrimental if HS is combined with traumatic brain injury (TBI), owing to the confined nature of the brain in the cranial cavity, the susceptibility to hypoxia and the well established detrimental effects of the associated intracranial pressure (ICP) changes [7].

Using a large animal model of combined TBI and HS, our group has previously demonstrated that early bolus resuscitation with FFP attenuates both lesion size and fluid extravasation into the brain compared to bolus resuscitation with 0.9% normal saline (NS). Using the same model, we now hypothesize that stepwise infusion speed increment resuscitation with FFP or NS will be superior to bolus in reducing brain lesion size and edema formation.

## Method

All the research was conducted in compliance with the Animal Welfare Act and other Federal statutes and regulations relating to animals and experiments involving animals. The study adhered to the principles stated in the Guide for the Care and Use of Laboratory Animals, Institute for Laboratory Animal Research (1996) and was approved by the appropriate Institutional Animal Care and Use Committees. All the procedures were performed under the supervision of a veterinarian.

A total 23 Female Yorkshire swine (40–50 kg; Tufts Veterinary School, Grafton, MA) were used and allowed three days for acclimatization prior to surgery. Of these, 20 animals were primarily used for and included in other studies [8],[9] with an identical protocol while the remaining three animals were included for the purpose of the present study. The aims of these other studies were to compare the effects of different resuscitation fluids on brain injury. The present study thus presents secondary use of animal experiments previously published as well as novel data from animals not previously published.

Food was withheld the night before surgery, but access to water was allowed. Preanesthesia was administered with an intramuscular injection of Tiletamine/Zolazepam (Telazol, 50 mg/ml), 8 mg/kg (Fort Dodge Animal Health, Fort Dodge IA) and atropine sulfate 1.5 mg. Animals were weighed and anesthesia was subsequently induced with inhalation of 4% inspired fraction isoflurane in 100% oxygen. Animals were intubated with a 7.0 mm cuffed endotracheal tube and put on ventilator support (Narkomed-M, North American Dr\ager, Telford PA) with a tidal volume of 10 ml/kg, peak pressure of 20 cm H_2_0 and a respiratory rate of 10 breaths per minute. No supplemental oxygen was administered following intubation. Tidal volumes and respiratory rate was adjusted to maintain a target end tidal PCO_2_ of 40 mmHg. Isoflurane was adjusted to 1%-3% inspired fraction for maintenance of anesthesia.

### Instrumentation and monitoring

After induction of anesthesia, a cutdown technique was used to access the right and left femoral arteries for invasive blood pressure monitoring (Eagle 4000 patient monitor, GE Marquette Piscataway NJ) and blood draw respectively. The left femoral vein was cannulated for fluid administration, whereas the right external jugular vein was used for the insertion of a pulmonary artery (PA) catheter. The PA catheter was used for measurements of cardiac output, pulmonary and central venous pressures as well as mixed venous oxygenation. A distal midline laparotomy was performed for the insertion of a cystostomy tube. Hemodynamic parameters (V9004 SurgiVet, Waukesha, WI), including cardiac output (Vigilance II Monitor, Edwards Lifesciences, Irvine CA), were recorded in five-minute intervals. The animal’s head was fixed in a custom made stereotactic frame with a mouthpiece affixed to the zygoma to prevent movement.

A 20 mm burr hole was made on the right side of the skull, next to the coronal and sagittal sutures over the frontal lobe to expose the dura. Bone was carefully removed so as not to disturb the dura and the underlying brain tissue. A catheter for intracranial pressure monitoring and monitoring of cerebral oxygenation (Integra Lifesciences, Plainsboro, NJ) was inserted through a bolt placed in a 2-mm burr hole on the left side of the skull, 10 mm lateral and 10 mm anterior to the bregma.

A computer-controlled cortical impact device was used for these experiments. Briefly, a 15 mm cylindrical impactor tip was mounted on an electronic motor, and the dynamics were precisely controlled to deliver 4 m/s velocity, 100 ms dwell time and 12 mm depth penetration. After impact, the burr hole was sealed with bone wax to prevent leakage of cerebrospinal fluid, and to eliminate any artifact in ICP monitoring.

### Hemorrhage and resuscitation protocol

**Figure 1:**
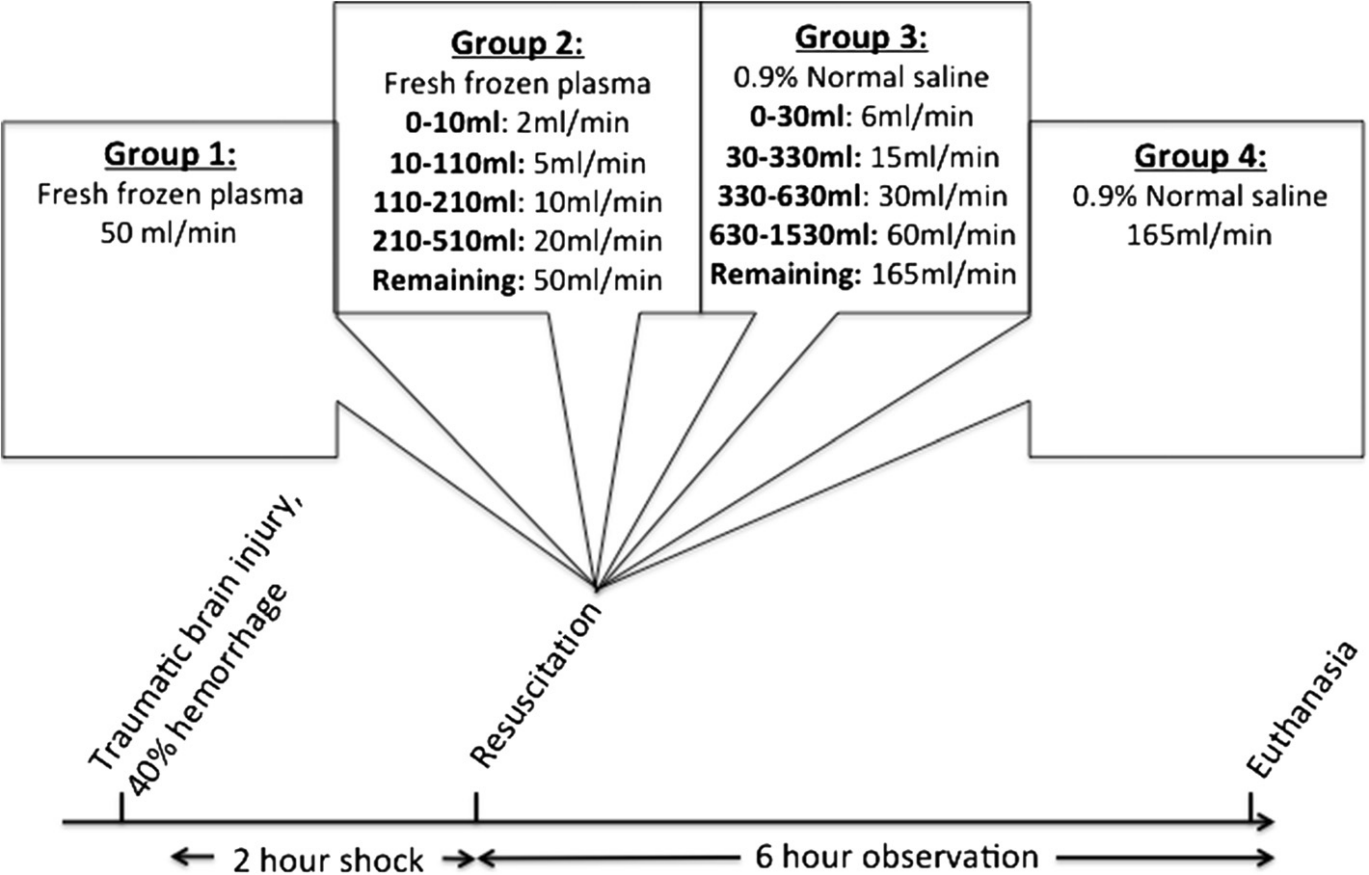
**Model overview.** Groups 1 and 4 were resuscitated with fixed infusion speeds as seen, whereas groups 2 and 3 were resuscitated with stepwise speed increments. Speeds were increased once the given fluid volumes depicted in the group boxes had been infused.

Model outline is provided in Figure 1. Total blood volume was estimated, and 40% of it was withdrawn through the femoral arterial catheter using a Masterflex pump, Model L/S Computerized Drive with a MF easy load II Pump-head, Model 77201–60 (Cole-Palmer, Vernon Hills, IL). Bleeding was started concurrent with TBI at a rate of 3.15% total blood volume/min and was captured in a Terumo blood collection bag (CPDA and AS-5). Isoflurane was decreased with the onset of hypotension. If MAP dropped < 30 mmHg, hemorrhage was briefly held and a small volume of saline was infused through the femoral venous line. Once the MAP reached 35 mmHg, saline infusion was stopped and hemorrhage was restarted. Using this protocol, MAP was maintained between 30 mmHg and 35 mm Hg until 40% of the estimated blood volume was withdrawn in a controlled fashion. Following hemorrhage, animals were left in shock for 120 minutes and MAP was maintained between 30–35 mmHg by titrating the dose of inhaled isoflurane. After the 2 hours of shock, animals were resuscitated as follows: Prior to the experiment, animals had been randomly assigned to 1 of 4 resuscitation groups (Figure 1): 1) bolus infusion with FFP 1*shed blood volume (n = 5) at 50 ml/min, 2) Stepwise infusion of FFP 1*shed blood with infusion speeds starting at 2 ml/min and gradually increasing to 50 ml/min (n = 6), 3) stepwise infusion of NS*3 shed blood volume with infusion speeds staring at 6 ml/min and gradually increasing to 165 ml/min (n = 6) and 4) bolus infusion of NS *3 shed blood volume at 165 ml/min (n = 5). Bolus resuscitation protocols were based on previous experiments [9]. The stepwise resuscitation protocols were the result of several pilot experiments suggesting an optimal hemodynamic response while minimizing resuscitation time and the extent of the brain injury. Fluids were infused into the femoral vein using a Masterflex pump. Animals were monitored for six hours post resuscitation and were kept warm (Bair Hugger Model 505; Arizant Healthcare Inc., Eden Praire, MN). Electrolytes were corrected as needed. Following the six-hour observation period, animals were euthanized by intravenous injection of sodium pentobarbital 100 mg/kg (Euthasol, Virbac Corp, Fort Worth, TX).

An arterial blood gas was analyzed at baseline, following 2 hours of shock and the 6-hour observation period.

### Resuscitation fluids

Normal saline (0.9% NaCl) was obtained from Hospira inc. (Lake Forest, IL) and stored at room temperature. FFP was isolated from healthy porcine donors. Briefly, whole blood was captured in a blood collection bag and centrifuged at 5000 g for 10 minutes at 4°C. The supernatant was further centrifuged at 5000 g for 10 minutes to extract the plasma which was stored at −80°C. It was thawed immediately prior to use.

### Calculation of brain infarction and swelling

Brains were removed following euthanasia and were sliced into 5 mm coronal sections. Slices were incubated in 2% 2,3,5-triphenyltetrazolium chloride (TTC) (SigmaChemical Co., St. Louis, MO) to assess the presence of nonviable tissue. Size of the lesion was measured with computer-assisted image analysis software ImageJ (NIH, Bethesda, MD). Brain swelling was calculated by comparing it to the uninjured hemisphere [(ipsilateral hemisphere’s volume/contralateral hemisphere’s volume) − 1] × 100 . True infarction volumes were corrected by the swelling factor.

### Derived variables

Stroke volume (SV) was calculated as Cardiac Output/Heart Rate. Systemic vascular resistance (SVR) was calculated as 80 × (mean arterial pressure-central venous pressure)/Cardiac Output.

### Statistical analysis

Data are presented as mean ± standard error of the mean. Brain lesion size and swelling as well as arterial blood gas parameters were compared using an unpaired t-test.

For the purpose of comparing post-resuscitation hemodynamic variables, following biostatistician consultation we used a linear mixed model to compare value means from end of resuscitation through the 6-hour observation period between groups while correcting for intra-group repeated measures. Based on these measurements, the model calculated an estimated mean value for the 6-hour timeframe in each group.

Sample size calculations were based on previously published data [9],[10] from the same model, and the group sizes were sufficient to detect a 50% difference between the groups for continuous variables (with variance of 25% within the group), with a power (1-β) = 90% and α = 0.05.

All statistical analysis was done using SPSS 20.0 (IBM Corp. Armonk NY).

Statistical significance was defined as p < 0.05.

## Results

### Arterial blood gas

Arterial blood gas values are listed in Table 1. At the 6-hour observation time point, fast infusion of FFP was associated with higher hemoglobin levels compared with stepwise FFP infusion (6.60 ± 0.44 g/dl vs. 5.12 ± 1.13 g/dl, p = 0.01) as well as higher lactate levels (2.94 ± 0.22 mmol/l vs. 2.04 ± 0.12 mmol/l, p = 0.01). No differences were found in the NS groups.

**Table 1:** Arterial blood gas values

	**Group**	**Baseline**	**Post 2 hour shock**	**6 hour observation**	p-value*
**pH**	FFP Fast	7.45 ± 0.02	7.37 ± 0.03	7.50 ± 0.02	0.84
	FFP Slow	7.47 ± 0.02	7.45 ± 0.01	7.49 ± 0.01	
	NS Fast	7.44 ± 0.01	7.41 ± 0.02	7.43 ± 0.02	0.21
	NS Slow	7.45 ± 0.01	7.43 ± 0.01	7.39 ± 0.03	
**pO2 (mmHg)**	FFP Fast	107.94 ± 6.37	97.26 ± 2.91	99.72 ± 7.55	0.99
	FFP Slow	94.38 ± 2.08	103.54 ± 8.68	99.72 ± 1.95	
	NS Fast	105.52 ± 4.08	99.50 ± 5.11	92.98 ± 5.02	0.10
	NS Slow	91.71 ± 2.41	97.67 ± 3.24	81.45 ± 3.70	
**pCO2 (mmHg)**	FFP Fast	36.94 ± 1.52	42.16 ± 1.50	38.26 ± 1.44	0.61
	FFP Slow	35.98 ± 1.69	31.92 ± 1.55	39.25 ± 1.18	
	NS Fast	34.36 ± 2.29	41.36 ± 3.95	37.24 ± 1.93	0.93
	NS Slow	38.52 ± 1.02	35.94 ± 1.74	37.49 ± 1.92	
**Hemoglobin (g/dl)**	FFP Fast	9.92 ± 0.43	10.74 ± 0.28	6.60 ± 0.44	**0.01**
	FFP Slow	9.42 ± 0.24	10.32 ± 0.14	5.12 ± 1.13	
	NS Fast	9.12 ± 0.18	10.06 ± 0.47	6.58 ± 0.29	0.10
	NS Slow	8.78 ± 0.25	10.02 ± 0.21	5.98 ± 0.23	
**Lactate (mmol/l)**	FFP Fast	1.60 ± 0.12	5.18 ± 1.18	2.94 ± 0.26	**0.01**
	FFP Slow	1.50 ± 0.21	3.64 ± 0.07	2.04 ± 0.12	
	NS Fast	1.36 ± 0.16	3.20 ± 0.50	1.48 ± 0.29	0.74
	NS Slow	1.44 ± 0.14	4.46 ± 1.44	1.34 ± 0.30	

Data presented as mean ± standard error of the mean.

*Denotes comparisons between slow and fast infusion of similar fluid types at the 6-hour observation time point. P-values in bold indicate significant differences.

### Systemic and pulmonary pressures

**Figure 2:**
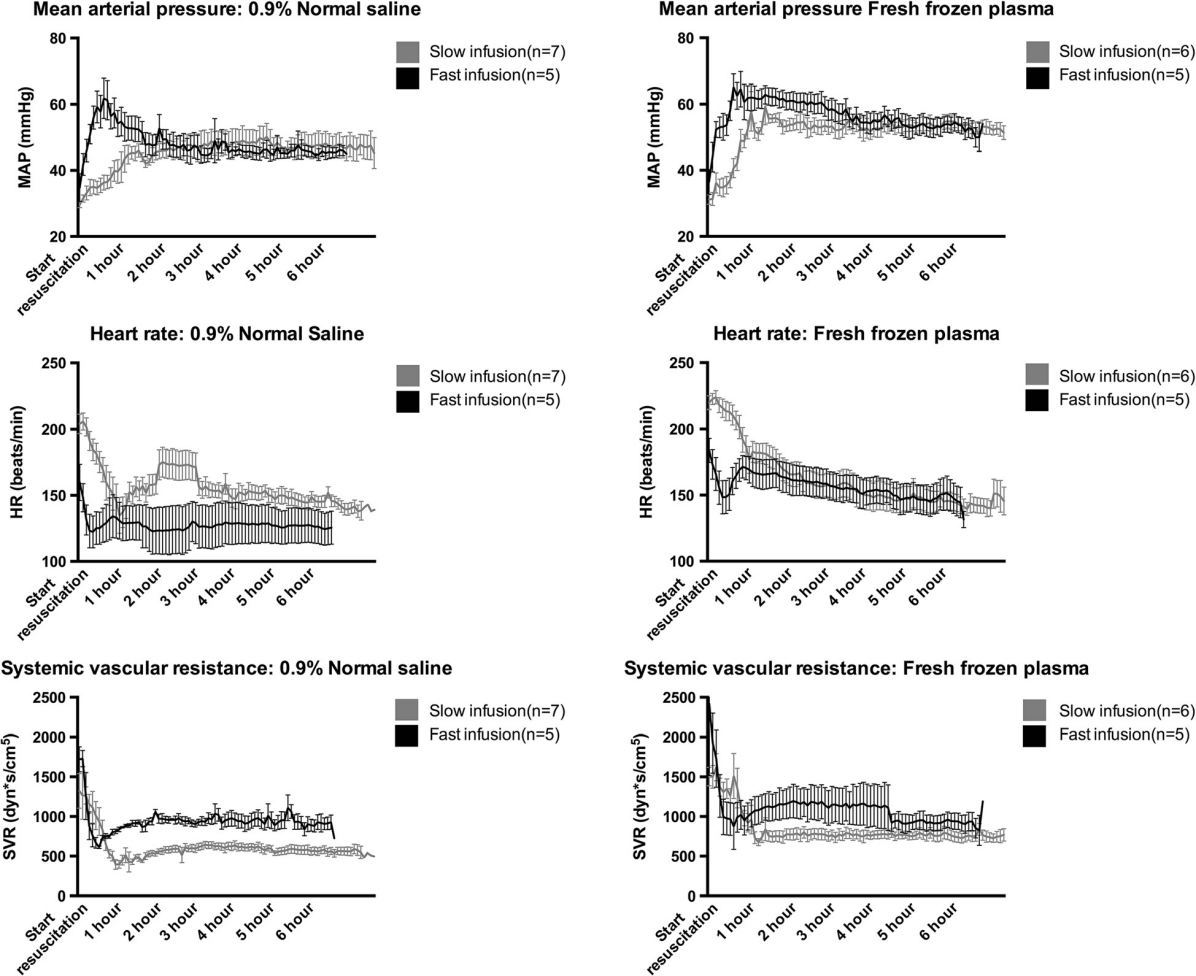
**Mean arterial pressure (top), heart rate (middle) and systemic vascular resistance (bottom) over the course of the 6-hour post resuscitation period.** Data presented as means ± standard error of the mean.

Hemodynamic variables are shown in Table 2 while Figure 2 depicts selected variables from start of resuscitation and over the course of the 6-hour observation period.

**Table 2:** Hemodynamic variables, intracranial pressure and brain oxygenation

	**Group**	**Baseline**	**Post 2 hour shock**	Post resuscitation and 6 hour observation*	**p-value#**
**Heart rate (Beats/min)**	FFP Fast	110.83 ± 9.83	182.67 ± 6.66	144.94 ± 7.45	0.27
	FFP Slow	112.20 ± 3.12	215.80 ± 8.84	156.83 ± 7.13	
	NS Fast	108.00 ± 10.33	177.00 ± 17.60	123.51 ± 6.70	**0.01**
	NS Slow	97.00 ± 4.11	204.67 ± 8.18	149.00 ± 6.16	
**MAP (mmHg)**	FFP Fast	73.83 ± 3.59	31.66 ± 0.0.99	55.90 ± 0.93	**0.05**
	FFP Slow	64.40 ± 2.69	34.20 ± 3.01	53.39 ± 0.86	
	NS Fast	77.50 ± 6.82	32.25 ± 0.95	52.93 ± 1.41	**0.01**
	NS Slow	59.17 ± 3.64	30.00 ± 0.52	46.92 ± 1.30	
**spO2 (%)**	FFP Fast	94.86 ± 0.40	92.00 ± 1.57	94.22 ± 0.52	0.06
	FFP Slow	94.00 ± 0.89	90.00 ± 2.63	92.79 ± 0.49	
	NS Fast	94.25 ± 0.48	92.50 ± 3.88	94.05 ± 0.34	**<0.01**
	NS Slow	94.00 ± 0.52	94.17 ± 1.19	91.37 ± 0.43	
**MPAP (mmHg)**	FFP Fast	19.33 ± 1.69	12.17 ± 3.16	15.12 ± 1.26	**<0.01**
	FFP Slow	16.00 ± 1.70	20.60 ± 4.82	23.11 ± 1.17	
	NS Fast	20.75 ± 1.32	14.50 ± 6.00	18.93 ± 0.85	0.66
	NS Slow	17.33 ± 1.82	14.67 ± 1.99	20.22 ± 1.16	
**CVP (mmHg)**	FFP Fast	2.50 ± 1.50	−3.83 ± 1.85	−0.07 ± 0.92	**0.03**
	FFP Slow	2.40 ± 1.81	−0.60 ± 2.34	2.85 ± 0.85	
	NS Fast	5.00 ± 0.71	0.01 ± 1.78	3.50 ± 1.08	0.90
	NS Slow	3.50 ± 1.25	−7.50 ± 8.56	3.31 ± 0.99	
**Cardiac Output (l/min)**	FFP Fast	5.17 ± 0.51	1.85 ± 1.13	4.81 ± 1.50	**0.03**
	FFP Slow	5.90 ± 0.27	2.38 ± 0.46	5.45 ± 1.14	
	NS Fast	5.65 ± 0.49	1.75 ± 0.15	4.37 ± 0.12	**<0.01**
	NS Slow	5.77 ± 0.55	2.05 ± 0.37	6.35 ± 0.10	
**ICP (mmHg)**	FFP Fast	7.33 ± 0.71	5.29 ± 0.97	9.10 ± 0.72	**0.02**
	FFP Slow	3.40 ± 1.36	1.80 ± 1.74	6.36 ± 0.68	
	NS Fast	5.75 ± 0.48	5.87 ± 1.63	9.39 ± 0.83	0.11
	NS Slow	4.00 ± 1.13	−0.33 ± 1.08	7.50 ± 0.76	
**Brain pO2 (mmHg)**	FFP Fast	14.80 ± 2.63	7.37 ± 1.48	10.42 ± 2.33	0.87
	FFP Slow	13.76 ± 2.68	6.20 ± 1.29	9.90 ± 2.28	
	NS Fast	12.00 ± 1.14	5.87 ± 1.63	10.32 ± 1.90	0.09
	NS Slow	13.87 ± 3.65	8.90 ± 3.96	14.54 ± 1.56	
**Stroke volume (ml/stroke)**	FFP Fast	47.51 ± 3.90	10.41 ± 0.94	33.26 ± 1.64	0.25
	FFP Slow	52.55 ± 1.50	11.48 ± 2.88	35.90 ± 1.54	
	NS Fast	53.15 ± 5.12	10.10 ± 0.96	42.93 ± 4.63	0.87
	NS Slow	58.86 ± 3.25	10.18 ± 2.09	41.92 ± 4.28	
SVR (dyn*s/cm^**5**^**)**	FFP Fast	1137.63 ± 3.34	1566.77 ± 163.67	1052.75 ± 65.90	**0.04**
	FFP Slow	850.13 ± 61.00	1280.60 ± 149.39	761.63 ± 22.42	
	NS Fast	1059.18 ± 163.17	1482.81 ± 51.77	921.40 ± 19.42	**<0.01**
	NS Slow	822.37 ± 107.35	1468.74 ± 132.93	573.41 ± 17.92	

SPB: Systolic blood pressure; MAP: Mean arterial pressure; MPAP: Mean pulmonary artery pressure; CVP: Central venous pressure; ICP: Intracranial pressure; SVR: Systemic vascular resistance. (*) Estimated marginal means by the statistical model throughout the 6-hour observation phase. (#) Comparisons of same fluid type estimated means using a linear mixed model. P-values in bold indicate significant differences.

In the FFP groups, fast resuscitation resulted in overall higher mean arterial pressures (MAP, 55.90 ± 0.93 mmHg vs. 53.39 ± 0.86 mmHg, p = 0.05) but similar central venous pressures (CVP). This was, however, associated with a higher systemic vascular resistance in the fast resuscitation group (SVR, 1052.75 ± 65.90 mmHg vs. 761.63 ± 22.42 mmHg, p = 0.04) as well as a lower mean pulmonary artery pressures (MPAP, 15.12 ± 1.26 mmHg vs. 23.11 ± 1.17 mmHg, p < 0.01).

In the NS groups, fast NS resuscitation resulted in an overall higher MAP (52.93 ± 1.41 mmHg vs. 46.92 ± 1.30, p = 0.01), but no difference in CVP. As in the FFP groups, this was associated with a higher SVR (921.40 ± 19.42 mmHg vs. 573.41 ± 17.92 mmHg, p < 0.01).

### Cardiac function

**Figure 3:**
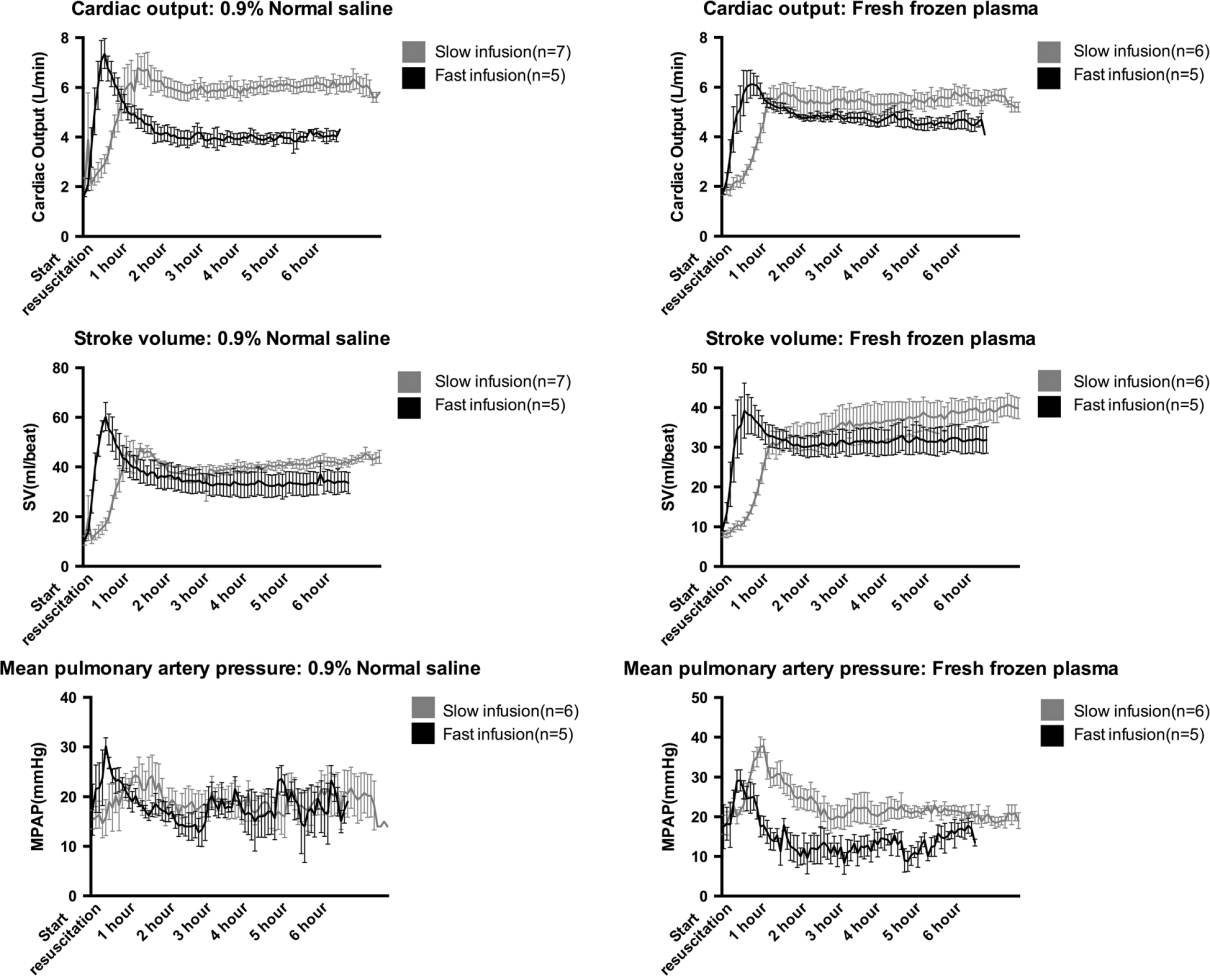
**Cardiac output (top), stroke volume (middle) and mean pulmonary artery pressure (bottom) over the course of the 6-hour post resuscitation period.** Data presented as means ± standard error of the mean.

Cardiac function is summarized in Table 2 and Figure 3. In both FFP and NS groups, fast resuscitation was associated with overall lower cardiac outputs (FFP: 4.81 ± 1.50 l/min vs. 5.45 ± 1.14 l/min, p = 0.03; NS: 4.37 ± 0.12 l/min vs. 6.35 ± 0.10 l/min, p < 0.01). No differences in stroke volume were observed.

### Intracranial pressure and brain oxygenation

**Figure 4:**
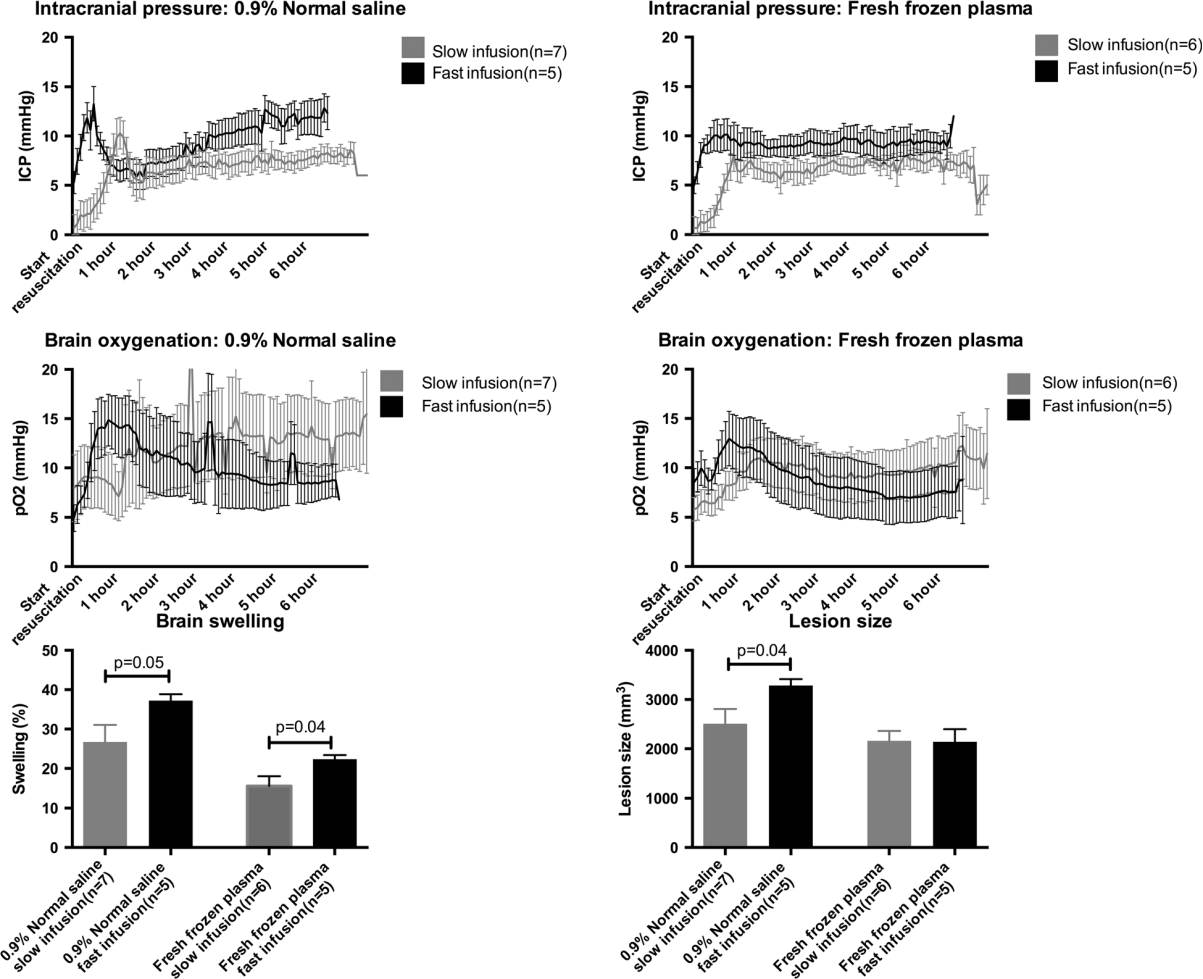
**Intracranial pressure (top) and brain oxygenation (middle) over the course of the 6-hour post resuscitation period.** Bottom bar graphs depict brain swelling (left) and lesion size (right) following the 6-hour observation period. Data presented as means ± standard error of the mean.

ICP and brain pO2 values are shown in Table 2 and Figure 4. Fast resuscitation with FFP resulted higher ICP (9.10 ± 0.72 mmHg vs. 6.36 ± 0.68 mmHg, p = 0.04) but no difference in brain oxygenation compared with stepwise resuscitation. No differences were observed in the NS groups.

### Lesion size and brain swelling

Lesion sizes and brain swelling is shown in Figure 4. Fast resuscitation with FFP resulted in increased brain swelling (22.36 ± 1.03% vs. 15.58 ± 2.52%, p = 0.04) but similar lesion sizes (2160.24 ± 202.56 mm^3^ vs. 2141.32 ± 256.27 mm^3^, p = 0.96) compared with stepwise FFP resuscitation. Fast resuscitation with normal saline resulted in increased brain swelling (37.24 ± 1.63% vs. 26.74 ± 1.33%, p = 0.05) as well as lesion size (3285.44 ± 130.81 mm^3^ vs. 2509.41 ± 297.44 mm^3^, p = 0.04).

## Discussion

In this study we found that bolus resuscitation with both FFP and NS resulted in adverse outcome compared with stepwise resuscitation. In the NS group, bolus resuscitation was associated with a significant increase in both brain swelling and lesion size, despite the fact that peripheral oxygenation (spO_2_) was higher in the bolus resuscitation group. Furthermore, a significant decrease in cardiac output coupled with an increase in systemic vascular resistance suggests that increased fluid extravasation may have compromised the resuscitative outcome.

In the FFP groups, these changes were mirrored although less pronounced. Bolus resuscitation was associated with increased fluid extravasation in the brain as well higher systemic vascular resistance, higher hemoglobin levels (suggesting less hemodilution and thus increased fluid extravasation) and lower cardiac output. These effects did, however, not appear to impact greatly on brain lesion size.

Overall, these findings are in line with previous reports suggesting that bolus resuscitation following hemorrhagic shock may be associated with adverse outcome [4]–[6]. Indeed, bolus infusion of crystalloids have been shown to increase bleeding and shorten survival times in a rodent model of hemorrhage following splenic injury compared to slow infusion [4]. Furthermore, while brisk infusion of normal saline may results in fluid extravasation in excess of 100 ml/min in humans, doubling the infusion time while maintaining equal fluid volume may result in a four time reduction of fluid extravasation rates, thus improving the volume load over time [11]. These results furthermore support the growing body of evidence suggesting favorable outcome when low volume resuscitation is used [12]–[14], but does raise the question of whether the beneficial effects are due to low volume or low infusion/perfusion pressures, or a combination.

Although this study was not designed for investigating the underlying pathophysiological mechanisms, some potential explanations may be considered. Following the classic line of thinking proposed by Starling [15], the greater increase in hydrostatic pressure during bolus resuscitation may drive a net fluid shift out of the vascular bed as oncotic pressures remain equal due to the equal amounts of identical fluids used. This is supported by findings indicating less early fluid extravasation in anesthetized compared to non-anesthetized patients receiving equal crystalloid volume loads [16]–[18], presumably due to vascular relaxation and concomitant lower perfusion pressures rather than choice of anesthetic agent [19]. In contrast, the degree of fluid extravasation following FFP infusion is largely unknown, but results from similar experiments using artificial colloids suggest that fluids extravasation may actually be increased during anesthesia [20]. The increased colloid osmotic pressure of FFP compared with NS may, however, counteract extravasation in the FFP groups with a resulting smaller difference in the hemodynamic response between bolus and slow resuscitation groups. Indeed, a differential effect on colloid osmotic pressure between crystalloids and artificial colloids has been demonstrated [21].

Recent evidence have challenged the classic Starling approach indicating a pivotal role of the semi-permeable layer formed by the endothelial glycocalyx [22]. This barrier coats the vascular endothelium and is comprised of membrane bound proteoglycans, glycoproteins and plasma proteins comprising a non-circulating intravascular layer of an estimated 700–1.500 ml [23],[24]. The integrity of the subglycolayx layer as well as the starling forces operating herein, more than intra and extravascular colloid osmotic and hydrostatic pressures seem determine net fluid shifts [25],[26]. It therefore follows that an intact and functional endothelial glycocalyx is pivotal for optimal resuscitative outcome.

Interestingly, volume loading with a concomitant increased stretch of the atrial walls release atrial natriuretic peptide (ANP), which directly induce shedding of the endothelial glycocalyx [27],[28]. Rapid reconstitution of the vascular volume may thus induce increased atrial stretching and ANP release, directly inducing glycococalyx shedding and increased fluid extravasation. Of note, recent evidence suggests that FFP may protect or even reconstitute the endothelial glycocalyx [29],[30], which may account for the less pronounced fluid extravasation observed in the FFP groups in this study.

Several limitations exist in this study and deserve to be acknowledged. We have chosen a 6-hour post-resuscitation observation time, which makes us unable to conclude on the long-term consequences of different resuscitation strategies. Indeed, the minor changes observed in ICP suggests that the observed brain swelling is likely not of clinical significance at this time point. The differential ICP and brain oxygenation trajectories evident from Figure 4 does, however, suggest that differences would only be exaggerated if the observation phase was extended.

The 6-hour observation time point does not allow us to conclude on the effects on clinically relevant outcome parameters such as neurofunctional status. The focus on brain lesion size, an end-point parameter of clear clinical relevance, still allows us to conclude on the clinical relevance of the differences observed in this model. A long-term survival model has been developed to address these important questions.

In line with this, the presented data does not allow us to conclude on the mechanistics underlying the observed differences. These issues are, however, the object of future more focused studies.

Secondly, neither NS nor FFP are usually employed as stand-alone resuscitative agents in the treatment of HS, bringing the clinical translatability of the model into question. Indeed, the use of packed red blood cells (PRBC’s) and platelets is standard of care in resuscitation of HS. We opted not to include PRBC’s and platelets in the resuscitation protocols since we wanted to examine the isolated effects of different resuscitation fluids rather than introduce the potential confounding effects of multiple fluids with unknown interactions. Indeed, the isolated effect of PRBC or platelet infusion speed on hemodynamics is unknown.

Third, the choice of bolus infusion speeds may be debatable. Indeed, we used maximum NS infusion speeds of 165/ml and FFP infusion speeds of 50 ml/min, which may appear higher than what is commonly used. It is, however, important to note that gravity alone can produce infusion crystalloid rates of 123 ml/min [31], pressure bags 257 ml/min [31] while rapid infusers such as the Level 1 (Level 1 technologies, Rockland, MA) and Rapid infusion system (Heamonetics Corp, Braintree, MA) can deliver infusion rates in excess of 800 ml/min [32].

In conclusion, in this study we found that bolus resuscitation with both NS and FFP following TBI and shock was associated with increased brain swelling in both groups as well as increased lesion size in the NS groups when compared to stepwise resuscitation. If these findings translate to humans it is concerning that widely used protocols such as the ATLS [3] still call for rapid infusion of crystalloids as the initial resuscitative adjunct in hypotensive patients.

## Competing interests

The authors declare that they have no competing interests.

## Authors’ contributions

MS, GJ and HA participated in study design, data analysis and manuscript preparation. PJ participated in study design, data analysis and manuscript preparation. HA obtained funding for the study. All authors read and approved the final manuscript.
